# TRAIL-R Deficient Mice Are Protected from Neurotoxic Effects of Amyloid-β

**DOI:** 10.3390/ijms231911625

**Published:** 2022-10-01

**Authors:** Giulia Di Benedetto, Chiara Burgaletto, Maria Francesca Serapide, Rosario Caltabiano, Antonio Munafò, Carlo Maria Bellanca, Rosaria Di Mauro, Renato Bernardini, Giuseppina Cantarella

**Affiliations:** 1 Section of Pharmacology, Department of Biomedical and Biotechnological Sciences, University of Catania, 95123 Catania, Italy; 2 Section of Physiology, Department of Biomedical and Biotechnological Sciences, University of Catania, 95123 Catania, Italy; 3 Section of Anatomic Pathology, Department of Medical and Surgical Sciences and Advanced Technologies “G.F. Ingrassia”, University of Catania, 95123 Catania, Italy; 4 Clinical Toxicology Unit, University Hospital of Catania, 95123 Catania, Italy

**Keywords:** TRAIL-R2, Alzheimer’s disease, apoptosis, neuroinflammation

## Abstract

TRAIL, a member of TNF superfamily, is a potent inducer of neuronal death. Neurotoxic effects of TRAIL appear mediated by its death receptor TRAIL-R2/DR5. To assess the role of TRAIL/TRAIL-R2 pathway in AD-related neurodegeneration, we studied the impact of the treatment with amyloid-β (Aβ) upon cell viability and inflammation in TRAIL-R-deficient mice (TRAIL-R^−/−^). Here, we demonstrate that the lack of TRAIL-R2 protects from death cultured TRAIL-R^−/−^ mouse embryonic hippocampal cells after treatment with either Aβ1-42 or TRAIL. Consistently, stereotaxic injection of Aβ1-42 resulted in blunted caspase activation, as well as in reduction of JNK phosphorylation and increased AKT phosphorylation in TRAIL-R^−/−^ mice. Moreover, the lack of TRAIL-R2 was associated with blunted constitutive p53 expression in mice that have undergone Aβ1-42 treatment, as well as in decrease of phosphorylated forms of tau and GSK3β proteins. Likewise, TRAIL-R2 appears essential to both TRAIL and Aβ-mediated neurotoxicity and inflammation. Indeed, hippocampi of TRAIL-R^−/−^ mice challenged with Aβ1-42, showed a slight expression of microglial (Iba-1) and astrocytic (GFAP) markers along with attenuated levels of IL-1β, TNF-α, NOS2 and COX2. In conclusion, the bulk of these results demonstrate that the constitutive lack of TRAIL-R2 is associated with a substantial reduction of noxious effects of Aβ1-42, providing further evidence on the prominent role played by TRAIL in course of Aβ-related neurodegeneration and confirming that the TRAIL system represents a potential target for innovative AD therapy.

## 1. Introduction

Alzheimer’s disease (AD) is the most common form of age-related dementia, characterized by an insidious onset of progressive cerebral atrophy and cognitive decline [1]. Distinctive neuropathological hallmarks of AD are represented by extracellular senile plaques of the amyloid-β (Aβ) peptide and by intracellular neurofibrillary tangles generated by hyperphosphorylated forms of the microtubule-binding protein tau [2]. Over the years, it has been demonstrated that many different factors such as genetics, free radicals, oxidative stress, mitochondrial dysfunction, or glutamate excitotoxicity may contribute to the pathogenesis and the progression of AD [3,4,5,6]. In addition, growing evidence suggest that AD pathogenesis is not restricted to the neuronal cell component, but it is significantly participated by an altered immune response [7,8].

In fact, neuronal damage, along with Aβ deposition, triggers activation of microglial and astrocytic cells also through the release of cytokines belonging to the Tumor Necrosis Factor (TNF) superfamily [9,10]. Among these, TNF-Related Apoptosis Inducing Ligand (TRAIL) is a potent proapoptotic molecule involved in either peripheral and central inflammatory processes [11,12]. TRAIL, released by activated glia [13], central nervous system (CNS)-infiltrating macrophages and damaged neurons, causes apoptosis in specific populations of cells in the CNS in course of neurodegenerative processes [14,15], including those related to ischemia [16,17], trauma [18], and Aβ accumulation [11,19]. TRAIL exerts its biological effects through a complex ligand–receptor system encompassing five cognate receptors [20]. Cytotoxic effects of TRAIL are mediated by two death domain receptors, TRAIL-R1/DR4 and TRAIL-R2/DR5 [21,22]. Interestingly, mice, unlike humans, harbour only the mTRAIL-R2/mDR5, which shows 79% of sequence homology with human DR5 [23]. In addition, whereas TRAIL is not detectable in the healthy human brain, and its death receptors are scarcely expressed [24], its expression is abundant in the AD brain [25], and, consistently, death receptors expression is in turn proportionally increased [11]. In vitro data [26] show that the blockade of the TRAIL receptor DR5 results in the prevention of Aβ-induced neurotoxicity, suggesting that DR5 is a key mediator of the TRAIL apoptotic pathway.

More recently, we have shown that immunoneutralization of TRAIL preserves cognitive behaviour, along with reduction of Aβ deposits and blunted expression of inflammatory molecules in the mouse brain [11].

In neuronal cell lines, DR5 expression is promoted by the tumour suppressor gene p53 [26], representing one main target gene, upregulated in response to DNA damage in different organs [27]. In addition, not only p53 directly regulates the expression of DR5, but also that of DR4, TRAIL-R3/DcR1 and TRAIL-R4/DcR2 [28,29,30,31].

Based upon this evidence, it appeared plausible to hypothesize a role of DR5 as a pivotal mediator of neuronal damage consequent to amyloid-related neuroinflammation. To address this hypothesis, the neurotoxic effects of Aβ have been assessed in a TRAIL-R deficient mouse (TRAIL-R^−/−^) [32], a strain which develops normally and with an intact immune system, without defects in thymic negative selection [33].

**Figure 1:**
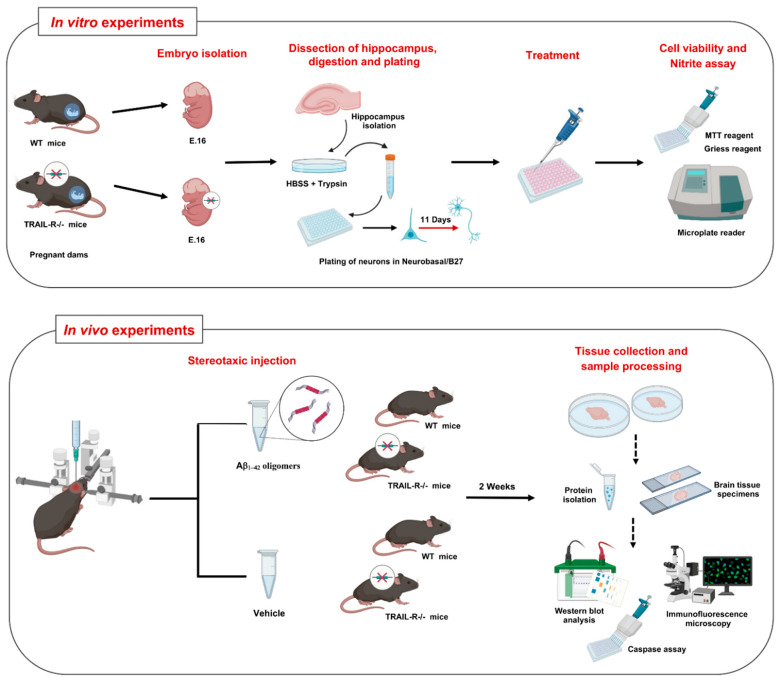
Experimental design for in vitro and in vivo experiments. Upper panel: Schematic representation of the main steps for the preparation of primary cultures of embryonic hippocampal cells derived from wild-type (WT) and TRAIL-R^−/−^ mice, including embryo isolation (16 embryonic day E.16), hippocampal dissection, enzymatic/mechanical digestion, plating of neurons and differentiation for 11 days. Differentiated neurons were treated with various compounds. After that, cell viability and nitrite assay were performed. Lower panel: The scheme summarizes the experimental design for in vivo experiments. Wild type (WT) and TRAIL-R^−/−^ mice were stereotaxically injected with Aβ1-42 oligomers and vehicle. After 2 weeks, animals were sacrificed, and tissues were collected to perform protein analysis using several techniques.

Thus, we verified this hypothesis both in in vitro studies, using primary cultures of hippocampal cells originated from TRAIL-R^−/−^ mice embryos, and in in vivo studies, wherein TRAIL-R^−/−^ mice were stereotactic injected in the dentate gyrus of the hippocampus with neurotoxic oligomers of Aβ1-42 with the aim to generate an AD phenotype (Figure 1).

## 2. Results

### 2.1. Amyloid Beta Neurotoxicity Is Significantly Attenuated in TRAIL-R^−/−^ Mouse Primary Neuronal Cells

**Figure 2:**
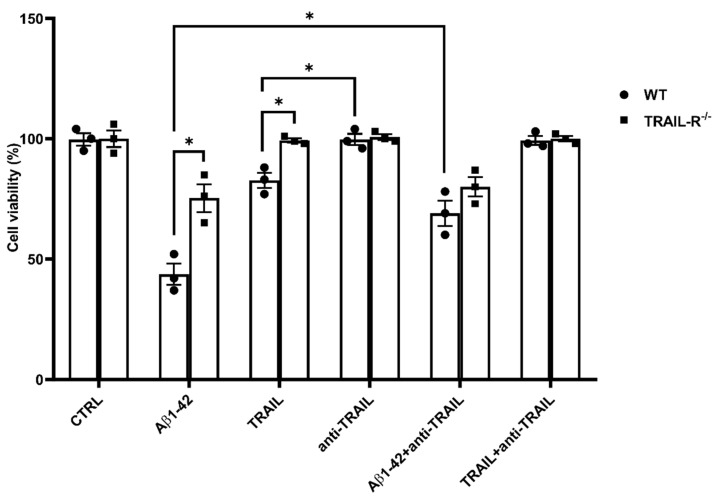
Amyloid beta neurotoxicity is attenuated in TRAIL-R^−/−^ mouse primary hippocampal cells. Cell viability of primary embryonic hippocampal neurons from WT and TRAIL-R^−/−^ mice, following 48 h treatment with Aβ1-42 (1 µM), TRAIL (100 ng/mL), anti-TRAIL antibody (1 µg/mL), or various combinations of the compounds. Vertical bars are means ± standard error mean (SEM). One-way ANOVA and the Bonferroni post-hoc test were used for statistical analysis. * *p* < 0.05.

To better understand the role of the TRAIL system in neurotoxic processes related to Aβ, in vitro cell viability experiments were performed on primary cultures of embryonic hippocampal cells derived from WT and TRAIL-R^−/−^ mice. Cells were challenged for 48 h with Aβ1-42 (1 µM), TRAIL (100 ng/mL), anti-TRAIL antibody (1 µg/mL), or various combinations of these substances (Figure 2). Aβ neurotoxicity observed in hippocampal cells from WT mice 48 h after treatmentwas significantly attenuated in cultured hippocampal cells from TRAIL-R^−/−^ mice. Moreover, the neurotoxic effect of Aβ1-42 on embryonic hippocampal cells of WT mice was significantly blunted by the treatment with a TRAIL neutralizing antibody (Figure 2). Overall, hippocampal cells from TRAIL-R^−/−^ mice showed a significantly higher resistance to death induced by both Aβ1-42 and TRAIL, as compared to cells from TRAIL-R-proficient mice.

### 2.2. TRAIL-R2 Is Required for p53 to Mediate Aβ-Related Neurotoxicity

**Figure 3:**
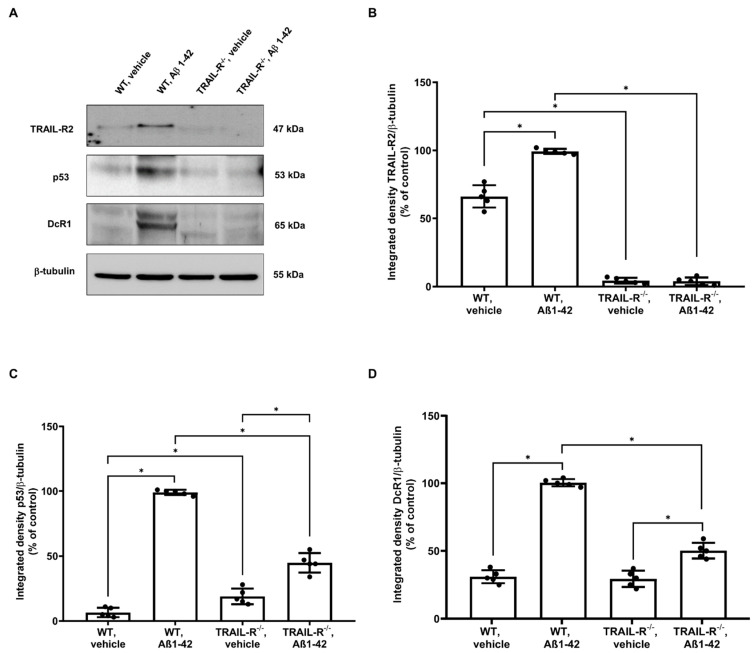
TRAIL-R2 is essential for p53 to mediate Aβ-related neurotoxicity. (**A**) Western blot for TRAIL-R2, p53, and DcR1 protein expression in the hippocampus of WT and TRAIL-R^−/−^ mice following stereotaxic infusion of oligomeric Aβ1-42 or vehicle. (**B**) Densitometric analysis of TRAIL-R2, (**C**) p53 and (**D**) DcR1 western blots. Data are expressed as means ± SEM. One-way ANOVA and the Bonferroni post-hoc test were used to determine statistical significance. * *p* < 0.05. N = 5 animals for each group.

To better understand the role of the TRAIL-R2 in Aβ-mediated neurotoxicity, we generated an AD phenotype in TRAIL-R^−/−^ mice, by performing stereotaxic injection of the neurotoxic oligomer Aβ1-42 into the dentate gyrus of the hippocampus. Since it is well known that the tumour suppressor gene p53 is involved in Aβ neurotoxicity [34,35,36] and that both TRAIL-R2 and DcR1 receptors are p53 target genes [29,30], we investigated the expression of these proteins in hippocampi from WT and TRAIL-R^−/−^ mice treated for two weeks with oligomeric Aβ1-42. Western blot analysis revealed that, while the expression of p53 and TRAIL-R2 was significantly enhanced in WT mice treated with Aβ1-42, p53 expression was blunted in TRAIL-R^−/−^ mice treated with Aβ1-42. While DcR1 expression was up-regulated in WT mice receiving Aβ1-42 treatment, it was down-regulated in TRAIL-R^−/−^ mice treated with Aβ1-42 (Figure 3). These results suggest that TRAIL-R2 may represent a crucial element for p53 to mediate Aβ-related neurotoxicity.

### 2.3. TRAIL-R^−/−^ Mice Show Reduced Caspase Activity after Challenge with Aβ1-42

**Figure 4:**
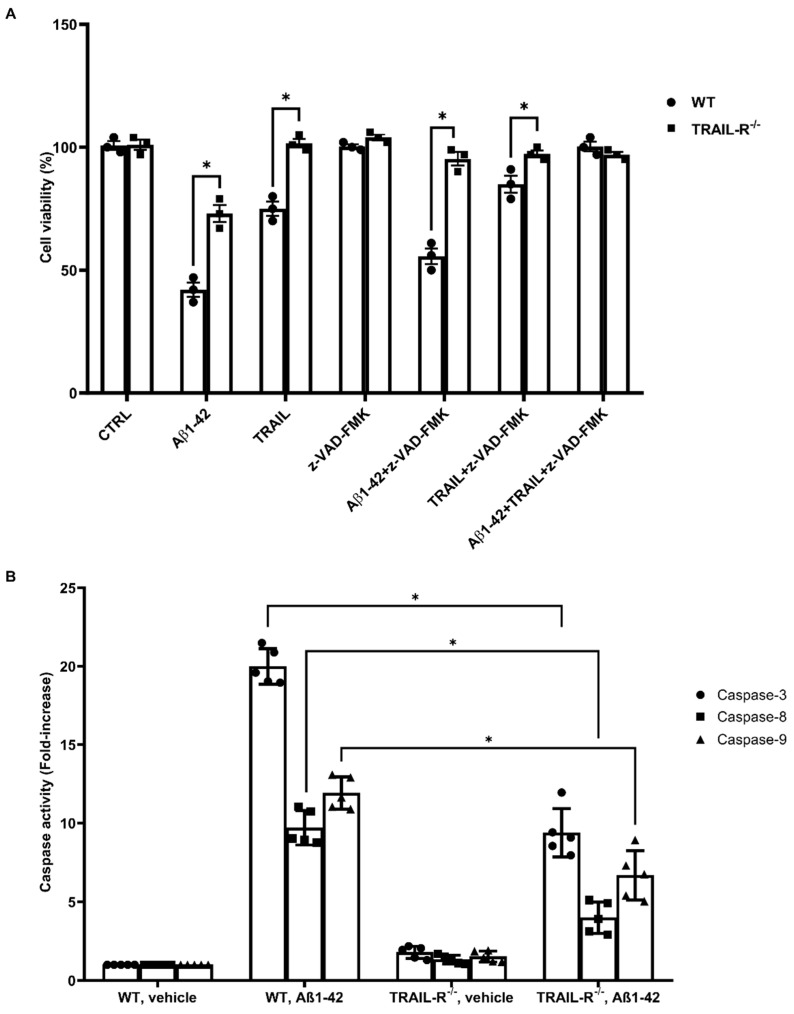
Reduced caspase activity in TRAIL-R^−/−^ mice challenged with Aβ1-42. (**A**) Embryonic hippocampal cell viability following 48 h treatment with Aβ1-42 (1 µM), TRAIL (100 ng/mL), z-VAD-FMK (2 µM), or various combinations of the compounds. Data are expressed as means ± SEM. One-way ANOVA and the Bonferroni post-hoc test were used to determine statistical significance. * *p* < 0.05. (**B**) Caspase-3, -8 and -9 activity in WT and TRAIL-R^−/−^ mice following stereotaxic infusion of oligomeric Aβ1-42 or vehicle. Data are expressed as means ± SEM. One-way ANOVA and the Bonferroni post-hoc test were used to determine statistical significance. * *p* < 0.05. N = 5 animals for each group.

Activation of the TRAIL-R2 is associated with the recruitment of caspase-8 and consequent activation of the caspase cascade, leading to cell death [37]. We studied this pathway in cultured embryonic hippocampal cells derived from WT and TRAIL-R^−/−^ mice (Figure 4A). Cells were incubated for 48 h with Aβ1-42 (1 µM), TRAIL (100 ng/mL), the pan-caspase inhibitor z-VAD-FMK (2 µM), or various combinations of these substances. While cell viability of TRAIL-R^−/−^ embryonic hippocampal cells challenged with Aβ1-42 or TRAIL was not affected by the treatment with the caspase inhibitor z-VAD-FMK, cells from WT mice, pre-treated with z-VAD-FMK and then treated with either Aβ1-42 or TRAIL, showed a significant increase of viability compared to those treated with Aβ1-42 or TRAIL alone.

To confirm the role of TRAIL-R2 in mediating Aβ neurotoxicity, we also assessed the activity of caspase-3, -8 and -9 in lysates of hippocampi from WT and TRAIL-R^−/−^ mice. Hippocampi from TRAIL-R^−/−^ mice treated with oligomeric Aβ1-42 showed decreased enzymatic activity of either caspase-8, as well as caspases-3 and -9, confirming data obtained with cultured hippocampal cells from TRAIL-R^−/−^ (Figure 4B).

These findings suggest that the lack of expression of the TRAIL-R2 results in a reduced caspases activation, leading to substantial protection from Aβ or TRAIL-induced neuronal death.

### 2.4. JNK and AKT Kinases Are Inversely Modulated in TRAIL-R^−/−^ Mice That Have Undergone Oligomeric Aβ1-42 Treatment

**Figure 5:**
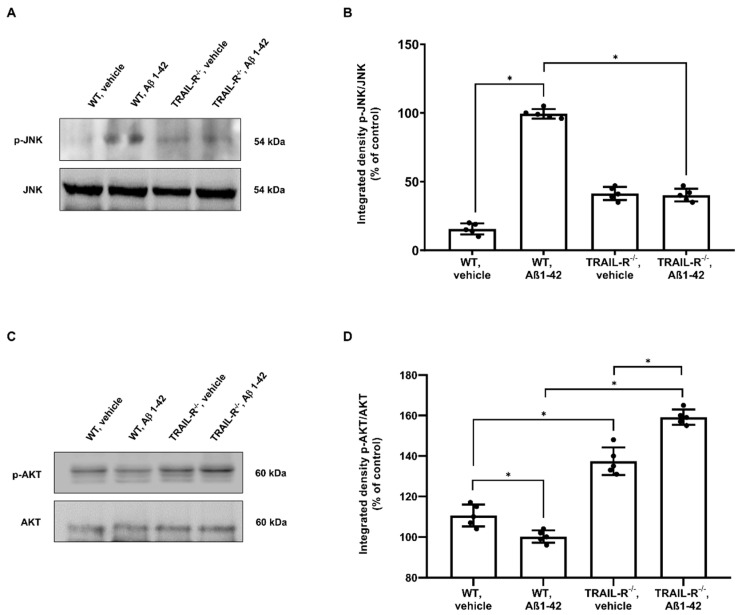
Inverse modulation of JNK and AKT kinases in TRAIL-R^−/−^ mice that underwent Aβ1-42 treatment. (**A**) Western blot analysis of p-JNK and (**C**) p-AKT in WT and TRAIL-R^−/−^ mice following stereotaxic infusion of oligomeric Aβ1-42 or vehicle. (**B**,**D**) are respective densitometric analysis of the western blots. Data are expressed as means ± SEM. One-way ANOVA and the Bonferroni post-hoc test were used to determine statistical significance. * *p* < 0.05. N = 5 animals for each group.

Downstream activity of death receptors involves a protein family named stress-cytokines-induced kinases, which include c-Jun N-terminal kinase (JNK), whose phosphorylation requires the cleavage of caspase-3, -8 and -9 [38,39,40]. Therefore, the involvement of this kinase was investigated in our model. Indeed, western blot analysis showed that JNK phosphorylation was significantly increased in WT mice treated with Aβ1-42, compared to TRAIL-R^−/−^ mice that underwent the same treatment (Figure 5A). The inhibition of JNK phosphorylation is in turn associated with activation of the AKT pathway, a central node in cell signalling downstream of growth factors, cytokines, and other cellular stimuli [41]. Western blot analysis confirmed that phosphorylation of AKT protein was, indeed, inversely correlated with JNK phosphorylation. In fact, whereas AKT phosphorylation was significantly blunted in WT mice that underwent Aβ1-42, it was significantly increased in TRAIL-R^−/−^ mice receiving the same treatment (Figure 5C).

### 2.5. Aβ1-42 Dependent GSK3β Activation and Tau Phosphorylation Are Attenuated in TRAIL-R^−/−^ Mice

**Figure 6:**
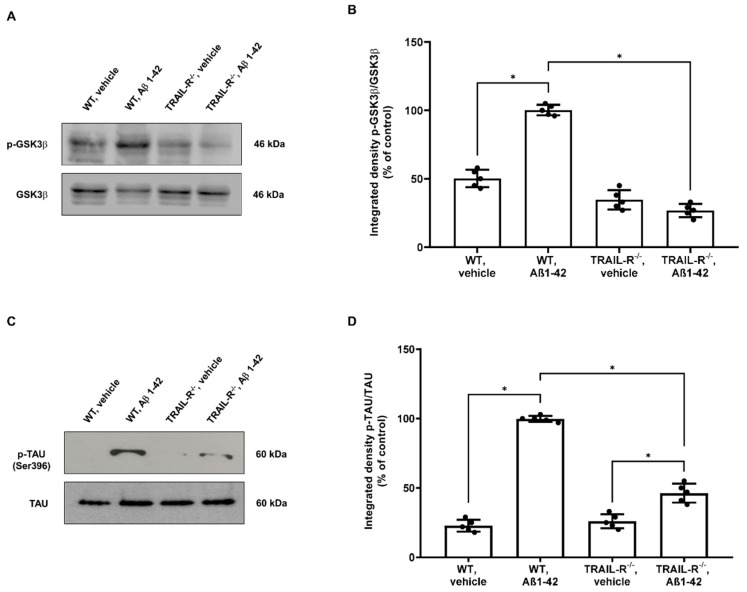
Phosphorylation of GSK3β and Tau is attenuated in TRAIL-R^−/−^ mice treated with oligomeric Aβ1-42. Western blot analysis of (**A**) p-GSK3β and (**C**) p-Tau in WT and TRAIL-R^−/−^ mice following stereotaxic infusion of oligomeric Aβ1-42 or vehicle. (**B**,**D**) are respective densitometric analysis of the western blots. Data are expressed as means ± SEM. One-way ANOVA and the Bonferroni post-hoc test were used to determine statistical significance. * *p* < 0.05. N = 5 animals for each group.

Glycogen synthase kinase-3β (GSK3β), an ubiquitously expressed serine/threonine kinase, plays a key role in the pathogenesis of AD, influencing Tau phosphorylation, Aβ production, neurogenesis and synaptic function [42]. In particular, it has been reported that GSK3β is phosphorylated in neurons challenged with Aβ or TRAIL [21]. For this reason, we checked whether the lack of TRAIL-R2 could be of help to better understand the role of TRAIL signalling pathway in this process. We, in fact, demonstrated that the expression of GSK3β phosphorylation was increased in WT mice that had undergone Aβ treatment whereas it was significantly decreased in TRAIL-R^−/−^ mice subjected to the same treatment. On the other hand, the lack of TRAIL-R2 did not affect the constitutive expression of GSK3β (Figure 6A).

Hyperphosphorylation of Tau protein is a process downstream the GSK3β phosphorylation [42] and is regarded as a major step in either the Aβ- or TRAIL-induced neuronal death [38]. Therefore, in order to assess whether the lack of TRAIL-R2 could impact upon Tau phosphorylation rate, the expression of both Tau and p-Tau (epitope Ser396) was analysed by western blot on hippocampal lysates from the same groups of animals mentioned above. TRAIL-R2 significantly affected tau phosphorylation which was highly increased in WT mice treated with Aβ1-42, while its phosphorylation was significantly attenuated in TRAIL-R^−/−^ mice treated with Aβ1-42 (Figure 6C).

### 2.6. Glial Response Is Blunted in TRAIL-R^−/−^ Mice Treated with Oligomeric Aβ1-42

In order to investigate the contribution of the TRAIL system to neuroinflammation, with regard to microglia and astrocyte activation, the expression of microglia and astrocyte markers along with the oligomeric Aβ1-42, were studied in the hippocampi from WT and TRAIL-R^−/−^ mice treated for 2 weeks with oligomeric Aβ1-42, administered stereotaxically.

**Figure 7:**
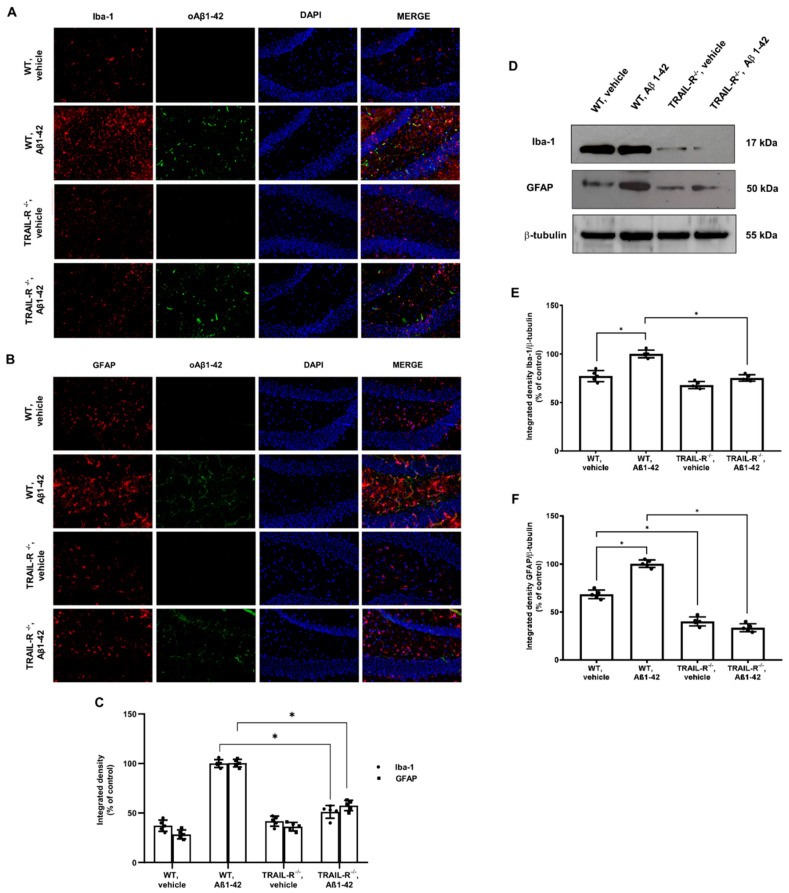
GFAP and Iba-1 are significantly reduced in the hippocampus of TRAIL-R^−/−^ mice treated with oligomeric Aβ1-42. Representative images (original magnification 20×) of the fluorescent immunohistochemical detection of (**A**) Iba-1 and oAβ1-42; (**B**) GFAP and oAβ1-42 expression in WT and TRAIL-R^−/−^ mice following stereotaxic infusion of oligomeric Aβ1-42 or vehicle. (**C**) Densitometric analysis of Iba-1 and GFAP immunofluorescence signal. (**D**) Western blot analysis of Iba-1 and GFAP in WT and TRAIL-R^−/−^ mice following stereotaxic infusion of oligomeric Aβ1-42 or vehicle. (**E**,**F**) are respective densitometric analysis of the western blots. Data are expressed as means ± SEM. One-way ANOVA and the Bonferroni post-hoc test were used to determine statistical significance. * *p* < 0.05. N = 5 animals for each group.

Immunofluorescence experiments revealed widespread glia activation in the brain of WT mice treated with Aβ1-42, as represented by high expression of astrocytic and microglial markers, respectively GFAP and Iba-1, whose localized in the vicinity of oligomeric Aβ1-42 immunopositive deposits. Interestingly, although oligomeric Aβ1-42 immunopositive deposits were unaffected in the hippocampus of TRAIL-R^−/−^ mice that had undergone Aβ1-42 treatment, the expression of both glial markers was significantly reduced in the hippocampus of the same animals (Figure 7A–C).

The immunofluorescence data for glial activation were confirmed by western blot analysis (Figure 7D–F).

These results demonstrate that the TRAIL system may play a pivotal role in glia activation occurring in course of neuroinflammation triggered by noxious stimuli such as oligomeric Aβ1-42.

### 2.7. Inflammatory Molecules Expression Is Reduced in TRAIL-R^−/−^ Mice Receiving Oligomeric Aβ1-42

As gliosis is a common pathological feature of neurodegenerative processes and it is intimately associated with neuroinflammation, we verified whether the TRAIL system could also modulate glial expression and release of pro-inflammatory mediators. Thus, either NOS2, COX2, IL-1β, and TNF-α proteins were analysed by western blot in hippocampal lysates from WT and TRAIL-R^−/−^ mice treated with oligomeric Aβ1-42.

**Figure 8:**
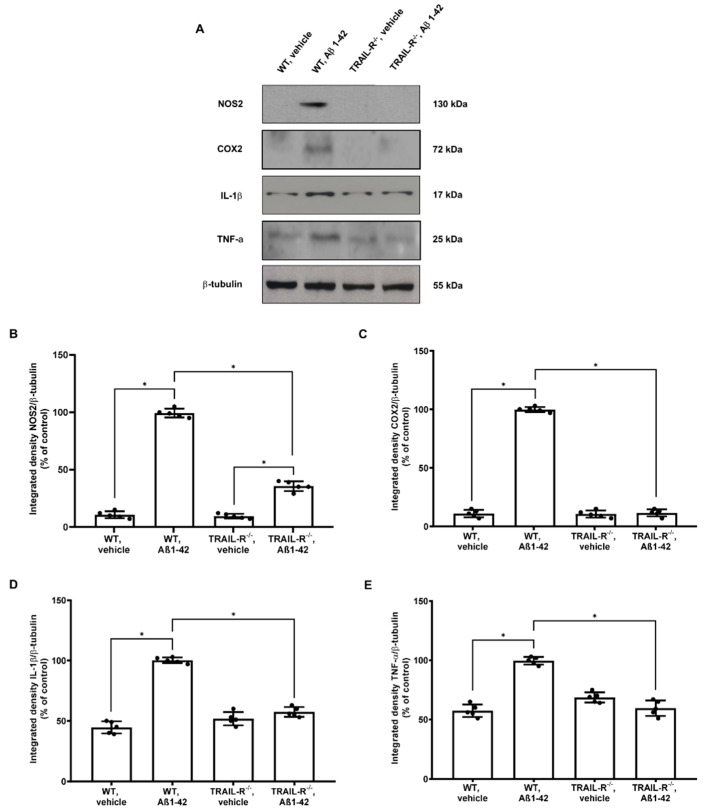
Inflammatory molecules expression is attenuated in TRAIL-R^−/−^ mice treated with Aβ1-42. (**A**) Western blot analysis of NOS2, COX2, IL-1β and TNF-α in WT and TRAIL-R^−/−^ mice following stereotaxic infusion of oligomeric Aβ1-42 or vehicle. (**B**–**E**) Densitometric analysis of western blots. Data are expressed as means ± SEM. One-way ANOVA and the Bonferroni post-hoc test were used to determine statistical significance. * *p* < 0.05. N = 5 animals for each group.

Results showed that, while NOS2, COX2, IL-1β, and TNF-α were expressed substantially in WT mice treated with Aβ1-42, their expression was significantly reduced in WT mice treated with vehicle and in TRAIL-R^−/−^ mice, suggesting that the TRAIL pathway provides a crucial contribution to neuroinflammation-related neurodegeneration (Figure 8A–E).

### 2.8. Nitrite Levels Are Significantly Attenuated in the Media from Embryonic Hippocampal Cell Cultures from TRAIL-R^−/−^ Mice Treated with Oligomeric Aβ1-42

Nitric oxide reactive derivatives have been implicated as non-specific inflammatory mediators of neuronal death in several neurodegenerative and neuroinflammatory conditions including AD [43]. In particular, it is reported that reactive nitrogen oxide species create a vicious cycle where they trigger Aβ deposition, which, in turn, induce immune cells activation [44].

**Figure 9:**
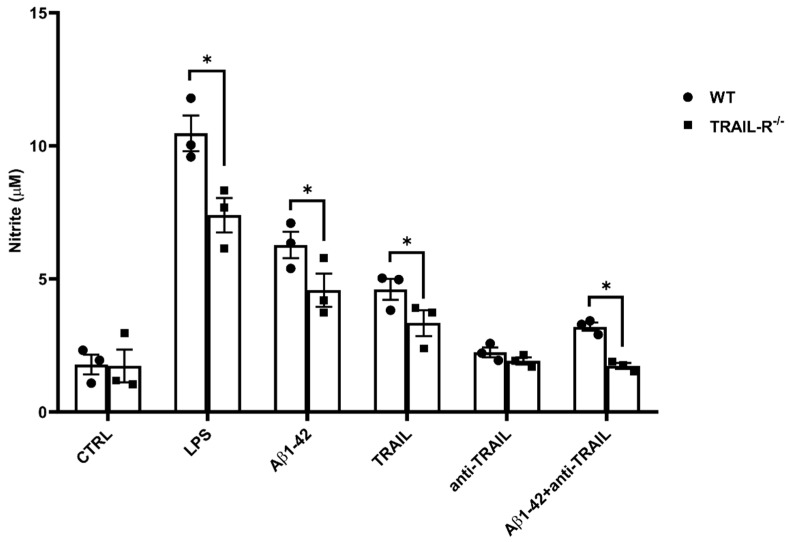
The lack of TRAIL-R2 is associated with a significant reduction of nitrite levels in the media of TRAIL-R^−/−^ mouse primary hippocampal cells treated with Aβ1-42. Nitrite levels in the media of embryonic hippocampal cells following 48 h treatment with LPS (10 µg/mL; positive control), Aβ1-42 (1 µM), TRAIL (100 ng/mL), anti-TRAIL (1 µg/mL) or Aβ1-42 plus anti-TRAIL. Data are expressed as mean ± SEM. One-way ANOVA and the Bonferroni post-hoc test were used to determine statistical significance. * *p* < 0.05.

With the purpose of validating, from a functional point of view, the reduced expression levels of NOS2 previously achieved by Western blot analysis in TRAIL-R^−/−^ mice, we investigated nitrite release in the media of embryonic hippocampal cells derived from WT and TRAIL-R^−/−^ mice challenged for 24 h with LPS (10 µg/mL) Aβ1-42 (1 µM), TRAIL (100 ng/mL), anti-TRAIL antibody (1 µg/mL), or various combinations of these compounds. Results revealed that the constitutive lack of TRAIL-R2 is associated with a significant reduction of nitrite levels in the various experimental conditions studied (Figure 9).

## 3. Discussion

Here, we studied the neurotoxic effects of Aβ in TRAIL-R^−/−^ mice [33] showing that, indeed, the TRAIL/TRAIL-R system plays a critical role in neuronal damage consequent to amyloid-related neuroinflammation.

Specifically, the role of TRAIL system in neurotoxic processes related to Aβ has been better defined in in vitro cell viability experiments performed on primary embryonic hippocampal cells derived from WT and TRAIL-R^−/−^ mice, which showed a significantly higher resistance to death induced by both Aβ1-42 or TRAIL. This is in line with previous findings showing that neutralization of TRAIL death pathway protects human neurons from Aβ toxicity [19].

It is well documented that the tumour suppressor gene p53 is a pleiotropic transcription factor that plays a crucial role in determining cell fate under certain conditions, including excitotoxicity, ischemic injury, ionizing radiation, and oxidative stress. p53 is constitutively present in many cell types including neurons and it is upregulated and activated via phosphorylation following these various insults resulting in the transactivation of different target genes which control both the processes of cell survival and death [45]. It is noteworthy that both TRAIL-R2 and DcR1 receptors are p53 target genes [29] and, while is clear that TRAIL-R2 upregulation depend upon p53 activation [46], the requirement of TRAIL-R2 during p53-mediated apoptosis remains still unclear. However, more recently, nuclear TRAIL-R2 has been shown to act as a negative regulator of p53, suggesting that it may heavily impact, for example, tumour growth [47]. Despite these findings, we found that the p53 expression was blunted in TRAIL-R^−/−^ mice treated with oligomeric Aβ1-42, suggesting that TRAIL-R2 contributes, in turn, to p53 mediation of Aβ-induced neurotoxicity. Consistently, p53 is downregulated in the white matter of DR5 null mice, a strain protected from radiation-induced cell death, supporting the role of p53-depending TRAIL-R2-related cell death in the CNS [48].

The downstream signal of TRAIL-R2 involves the engagement of the caspase cascade that induces apoptosis [37]. As a matter of fact, in our experiments the lack of TRAIL-R2 expression correlates with reduced activity of the caspase cascade, leading to effective protection from Aβ-induced neuronal death. Moreover, it is well documented that Aβ is able to induce the expression of TRAIL both in vitro [19] and in vivo [11] and that both TRAIL and Aβ may affect different pathways, including the stress-cytokines-induced kinase, such as JNK [38,49], as well as the serine/threonine kinase AKT, known to regulate numerous processes, including cell survival, growth, and apoptosis [50]. Following treatment with Aβ, we found lower levels of JNK phosphorylation in the hippocampi of TRAIL-R^−/−^ mice and, on the other hand, increased levels of AKT phosphorylation. These results corroborate the concept that the TRAIL-R2 may represent an essential element to trigger and fuel the cascade of events related to neurodegeneration in this model. As a proof, the lack of TRAIL-R2 is associated with failure in initiating the complex intracellular signalling machinery set into motion by Aβ-depending on TRAIL neurotoxicity.

In this complex intracellular signalling machinery, GSK3β represents one of the main enzymes responsible for hyperphosphorylation of the tau protein, which is a typical hallmark of AD-related neuroinflammation and neurodegeneration [42]. Both Aβ [49] or TRAIL [38], induce either phosphorylation of JNK and dephosphorylation of AKT, events connected with increased GSK3β phosphorylation, and subsequently Tau hyperphosphorylation [38]. Consistently, we found that, despite the significant Aβ-dependent increase of GSK3β phosphorylation and consequent Tau hyperphosphorylation occurring in the hippocampus of WT animals, mice lacking the TRAIL-R2 did not exhibit the same phosphorylation pattern. These data endorse the engagement of TRAIL-R2 as a critical event for the neurodegenerative process driven by Aβ via Tau hyperphosphorylation.

Moreover, TRAIL is known to induce gliosis, another typical feature of AD brain pathology [51]. In our hands, the immunofluorescence signal for GFAP and Iba-1 markers was negligible in the brain of TRAIL-R^−/−^ animals challenged with Aβ oligomers, supporting the hypothesis that the TRAIL system plays a pivotal role in sustaining activation of glia to fuel the neuroinflammatory machinery. Such increased expression of activated microglia and astrocytes is consistent with abundant expression and release of inflammatory mediators, such as NOS2, COX2, IL-1β, and TNF-α [52,53]. It is noteworthy that a TRAIL-neutralizing treatment results in a significant decrease of both cellular and soluble factors contributing to brain inflammation [11], suggesting that TRAIL is a coordinating molecule in the inflamed brain, as demonstrated, for example, by its capability to control peripheral immunocytes trafficking into the brain of 3xTg-AD mice [54]. This is in line with our results which demonstrate that in TRAIL-R^−/−^ mice that underwent Aβ oligomers treatment, the lack of TRAIL death receptors protects the brain from the detrimental effects mediated by gliosis and from increased expression of the molecular mediators of inflammation mentioned above.

## 4. Materials and Methods

### 4.1. Animals

TRAIL-R^−/−^ animals were kindly provided by Prof. H. Walczak, Dept of Cancer Biology, CRUK-UCL Cancer Ctr., UCL Cancer Inst., London, United Kingdom. TRAIL-R^−/−^ animals and wild-type (WT) littermates in the B6 background were maintained on a 12 h light/dark cycle in temperature and humidity-controlled rooms, and food and water were available ad libitum. All experiments were approved by the Italian Ministry of Health (authorization n.86/2015 PR) and conducted following the European Community directive guidelines for the use of animals in laboratory (2010/63/EU) and the Italian law (D.Lgs. 26/2014).

### 4.2. Preparation of Aβ1-42 Oligomers

Aβ1–42 oligomers were generated as the previously described method [55]. Briefly, the Aβ1–42 lyophilized peptide (Sigma-Aldrich, St. Louis, MO, USA) was initially dissolved in 1,1,1,3,3,3-hexafluoro-2-propanol (HFIP; Sigma-Aldrich) to a final concentration of 1 mM and incubated at room temperature for 2 h. The peptide solution was aliquoted and dried in the fume hood. Traces of HFIP was removed under vacuum in a SpeedVac centrifuge (800× *g*, RT), and the thin clear peptide film was stored over desiccant at −80 °C. For aggregation, the aliquoted peptide film was dissolved in dimethyl sulfoxide (DMSO) to 5 mM. The peptide in DMSO was diluted directly into sterile phosphate buffered saline (PBS, 1X) at 100 μM and incubated at 4 °C for 12 h to make the oligomeric form of Aβ1–42. Following incubation, Aβ1-42 samples were immediately used for the cell treatment or aliquoted and stored at –20 °C until their use.

### 4.3. Western Blot Analysis of Aβ1–42 Oligomers

Aβ samples were resolved by Tris-tricine PAGE (Invitrogen Corporation, Waltham, MA, USA) under nondenaturing/nonreducing conditions, and then transferred onto a nitrocellulose membrane (Amersham Biosciences, Buckinghamshire, UK). Membranes were blocked for 1 h, at room temperature (RT), in a solution of 5% non-fat dry milk in Tris-buffered saline containing 0.1% Tween-20 before incubation overnight at 4 °C with the mouse monoclonal antibody 6E10 (1:1000; Covance, Princeton, NJ, USA), which recognizes an epitope within residues 1–17 of human Aβ. Membranes were washed 3 times with Tris-buffered saline containing 0.1% Tween-20 and then incubated with the horseradish peroxidase-conjugated Ig anti-mouse antibody (1:2000; GE Healthcare, Milan, Italy) at RT for 1 h. Protein bands were visualized by enhanced chemiluminescence (Thermo Fisher Scientific, Waltham, MA, USA) and scanned with the iBright FL1500 Imaging System (Thermo Fisher Scientific) (see Supplementary Figure S1).

### 4.4. Experimental Groups and Drug Administration

Twenty TRAIL-R^−/−^ and 20 wild-type C57BL/6J male mice were enrolled at nine months of age and four study groups were used: (i) Wild-type plus vehicle; (ii) Wild-type plus Aβ1-42; (iii) TRAIL-R^−/−^ plus vehicle; and (iv) TRAIL-R^−/−^ plus Aβ1-42.

As previously described [56], mice were bilaterally injected with either oligomeric Aβ1–42 or with an equal volume of buffer solution (vehicle). Mice were positioned on a stereotaxic frame and a Hamilton syringe with a 29G needle was implanted into the dentate gyrus (DG) of the hippocampus using the following stereotaxic coordinates from the bregma: anterior-posterior (AP), −2.00 mm; medial-lateral (ML), ±1.3 mm; dorsal-ventral (DV), −2.2 mm. The ± sign preceding the ML value indicates left and right direction from the centre. The stereotaxic injector pump was activated to pump 4 µL of Aβ1-42 (100 µM) or an equal volume of vehicle into the DG at a rate of 0.5 µL/min. When the infusion was completed, the needle remained in place for an additional minute to minimize backflow of solution out of the injection site. Then, the needle was moved to the other side of the brain and injection was repeated. Animals were sacrificed after 2 weeks (Figure 1).

### 4.5. Primary Cultures of Mouse Hippocampal Neurons

Sixteen embryonic day mice (E.16) were obtained from surgically sacrificed pregnant mice and the hippocampus was separated under surgical stereomicroscope. Separated tissues were isolated and dissociated by mechanical/enzymatic dispersion. Cells were plated at a density of 1.5 × 10^5^ cells/cm^2^ in Neurobasal medium (Invitrogen Corporation) supplemented with 2% B27 (Invitrogen Corporation), 0.5 mM L-glutamine, and 50 U/mL penicillin/streptomycin (Invitrogen Corporation). Three days after plating, 50% of the medium was changed with fresh medium and subsequently 50% of the medium was changed twice a week, until 11 days in vitro. To inhibit glial cell outgrowth, cytosine arabinoside (1 μM) was added at the moment of media change (Figure 1).

### 4.6. Cell Viability Assay

Cell viability was determined by 3-[4,5 dimethylthiazol-2-yl]-2,5-diphenyltetrazolium bromide assay. At the end of each treatment, cell viability was measured by the reduction of 3-[4,5 dimethylthiazol-2-yl]-2,5-diphenyltetrazolium bromide solution (0.5 mg/mL). The solution was removed after 3 h of incubation at 37 °C and dimethylsulfoxide was added to obtain cell lysis and solubilization of blue formazan crystals resulting from MTT reduction by viable cells’ mitochondrial activity. Optical density of the blue formazan was measured at 570 nm with a VarioskanTM Flash Multimode Reader (Thermo Fisher Scientific).

### 4.7. Free-Floating Fluorescence Immunohistochemistry

Mice were deeply anesthetized by intraperitoneal (i.p.) injection of Zoletil 100 (40 mg/kg) (Virbac S.r.l., Milan, Italy) and perfused transcardially with 4% paraformaldehyde (PFA) solution in 0.1 M phosphate buffer (PBS; pH 7.4). The brains were removed, post-fixed overnight in the same 4% PFA and then transferred into a 30% sucrose in PBS as cryo-protective solution at 4 °C for 2–3 days. Serial 25 μm frozen hippocampal sections of the brains were cut and subjected to immunohistochemical assay. Briefly, free-floating sections were washed three times in PBS and then blocked at room temperature for 1 h in 5% normal goat serum (NGS) in PBS. They were then incubated overnight at 4 °C with the following antibodies: a mouse monoclonal anti-Iba1 (1:200, Abcam, Cambridge, UK) as microglial marker and a mouse anti-GFAP antibody (Cell Signaling Technology, Inc., Danvers, MA, United States). For fluorescence visualization, after washing in PBS three times for 5 min each, sections were incubated in the dark for 1 h at room temperature with the corresponding fluorescent-labelled secondary antibodies: Alexa Fluor 546 donkey anti-mouse IgG (1:500, Thermo Fisher Scientific) and Alexa Fluor 488 goat anti-mouse IgG (1:500, Thermo Fisher Scientific). Finally, sections were washed in PBS three times for 5 min each and mounted on gelatinated slides. Digital images were captured with a Zeiss Observer.Z1 microscope equipped with the Apotome.2 acquisition system (Zeiss, Oberkochen, Germany).

### 4.8. Protein Extraction

Freshly isolated hippocampal tissues were lysed in a buffer containing 150 mM NaCl, 50 mM Tris–HCl (pH 7.5), 5 mM EDTA, 1 mM Na_3_VO_4_, 30 mM sodium pyrophosphate, 50 mM NaF, 1 mM acid phenyl-methyl-sulphonyl-fluoride, 5 µg/mL aprotinin, 2 µg/mL leupeptin, 1 µg/mL pepstatin, 10% glycerol, and 0.2% Triton X-100. The homogenates were then centrifuged at 14,000 rpm for 10 min at 4 °C and supernatants were collected. Protein concentration of the supernatant was determined by the Bradford method [57].

### 4.9. Western Blot Analysis

Equal amounts of proteins (50 µg) were separated by 8–12% SDS-PAGE gels and transferred onto Hybond ECL nitrocellulose membranes (Amersham Life Science, Buckinghamshire, UK). The membranes were blocked with 5% non-fat dry milk in PBST for 1 h at RT and were then probed overnight at 4 °C on orbital shaker with the following appropriate primary antibodies: rabbit anti-DR5 (1:500; Abcam, Cambridge, United Kingdom), mouse anti-p53 (1:1000; Cell Signaling Technology, Inc.), rabbit anti-DcR1 (1:1000; Abcam), mouse anti-p-JNK (1:500; Santa Cruz Biotechnology Inc., Dallas, TX, USA), mouse anti-JNK (1:500; Santa Cruz Biotechnology), rabbit anti-p-AKT (1:1000; Cell Signaling Technology, Inc.), rabbit anti-AKT (1:1000; Cell Signaling Technology, Inc.), mouse anti-p-GSK3β (1:500; Becton Dickinson, Franklin Lakes, NJ, USA), mouse anti-GSK3β (1:500; Santa Cruz Biotechnology Inc.), mouse anti-p-TAU (Ser396) (1:250; Santa Cruz Biotechnology Inc.), rabbit anti-TAU (1:500; Santa Cruz Biotechnology Inc.), mouse anti-Iba-1 (1:500; Abcam), mouse anti-GFAP (1:500; Cell Signaling Technology), rabbit anti-NOS2 (1:500; Santa Cruz Biotechnology Inc.), mouse anti-COX2 (1:500; Santa Cruz Biotechnology Inc.), rabbit anti-IL-1β (1:250; Santa Cruz Biotechnology Inc.) and rabbit anti-TNF-α (Novus Biologicals, Centennial, CO, USA). Beta-tubulin (Santa Cruz Biotechnology Inc.) primary antibody was used as an internal control to validate the right amount of protein loaded in the gels. Then the membranes were washed with PBS-T and probed with the appropriate horseradish peroxidase-conjugated secondary antibodies (GE Healthcare) for 1 h at room temperature in 5% non-fat dry milk. After washing with PBS-T, protein bands were visualized by enhanced chemiluminescence (Thermo Fisher Scientific) and scanned with the iBright FL1500 Imaging System (Thermo Fisher Scientific). Densitometric analysis of band intensity was performed with the aid of ImageJ software (developed by NIH, freeware, available online: https://imagej.nih.gov/ij/, accessed on 25 July 2022).

### 4.10. Caspase Colorimetric Assay

To investigate the activation of caspase-3, -8 and -9, the Caspase Colorimetric Substrate Set II Plus kit (BioVision Inc., Milpitas, CA, USA) was used and the analysis was performed as previously described [38]. In brief, protein extracts (100 µg) of hippocampi from WT and TRAIL-R^−/−^ mice stereotaxically injected with oligomeric Aβ1-42 or vehicle were added to a reaction buffer containing a p-nitroanilide-labelled specific caspase substrate (Caspase-3 Substrate, Ac-DEVD-pNA; Caspase-8 Substrate, Ac-IETD-pNA; Caspase-9 Substrate, Ac-LEHD-pNA) and incubated for 2 h at 37 °C. Relative caspase activity was measured as optical density at 405 nm in a microplate reader (Bio-Rad Laboratories, Inc., Milan, Italy). Fold-increase in caspase activity was determined by comparing results with the level of the uninduced control.

### 4.11. Nitrite Assay

Nitric oxide production in the primary embryonic hippocampal neurons was assessed by the Griess method, as previously described [13]. Briefly, after 24 h treatments, 100 μL aliquots of culture supernatants were incubated with 100 μL of Griess reagent (1% sulphanilamide, 0.1% N-(1-naphtil)ethyl-enediamine dihydrochloride and 5% of phosphoric acid) at room temperature for 20 min. Optical density at 540 nm was determined using a microplate reader (Bio-Rad Laboratories). The nitrite concentration was determined from a sodium nitrite standard curve.

### 4.12. Statistical Analysis

Data were analysed by the one-way analysis of variance (ANOVA) test, followed by the Bonferroni post-hoc test. Data were represented as means ± standard error mean (SEM). Significance was set at a *p* < 0.05. Graph design and statistical analyses were carried out with the dedicated software GraphPad Prism (La Jolla, CA, USA).

## 5. Conclusions

Overall, our results demonstrated a pivotal role of the TRAIL-R2 in the pathogenesis of Alzheimer’s disease in a murine model. We showed that the lack of the TRAIL-R2 is associated with substantial restraint of the noxious effects of Aβ, providing genetically assessed evidence that the TRAIL/TRAIL-R system activated during neuroinflammatory processes is responsible for Aβ-induced neurotoxicity. Based on these results, the TRAIL system may be envisioned as a potential candidate target for effective therapeutic intervention in AD.

## Data Availability

The data presented in this study are available from the corresponding author on reasonable request.

## Supplementary Materials

The supporting information can be downloaded at: https://www.mdpi.com/article/10.3390/ijms231911625/s1.
