# Supplementary material for: TRAIL-R Deficient Mice Are Protected from Neurotoxic Effects of Amyloid-β
Source: Int J Mol Sci. 2022 Oct 1;23(19):11625. doi: 10.3390/ijms231911625 (PMC9569968; doi:10.3390/ijms231911625)
Supplement: Supplementary file 1 [file ijms-23-11625-s001.zip › ijms-1914073-supplementary.pdf]

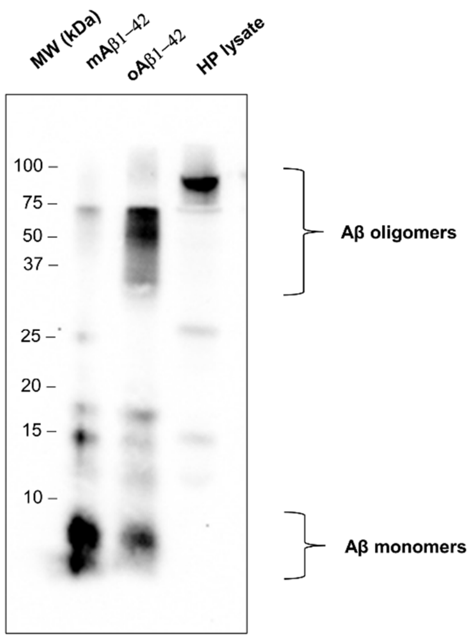

Supplementary Figure S1. Western blot for β-amyloid. Lane 1: Molecular weight marker; Lane 2: 1μg of the monomeric Aβ1-42 (mAβ1-42) peptide; Lane 3: 1μg of the oligomeric Aβ1-42 (oAβ1-42) peptide; Lane 4: 50μg of the hippocampal (HP) lysate.
